# Predicting Success in the Embryology Lab: The Use of Algorithmic Technologies in Knowledge Production

**DOI:** 10.1177/01622439211057105

**Published:** 2021-11-15

**Authors:** Alina Geampana, Manuela Perrotta

**Affiliations:** 1 Department of Sociology and Policy, School of Social Sciences and Humanities, Aston University, Birmingham, United Kingdom; 2 Department of People and Organisations, School of Business and Management, Queen Mary University of London, United Kingdom

**Keywords:** algorithm, embryology, IVF, knowledge, laboratory, standardization

## Abstract

This article analyzes local algorithmic practices resulting from the increased use of time-lapse (TL) imaging in fertility treatment. The data produced by TL technologies are expected to help professionals pick the best embryo for implantation. The emergence of TL has been characterized by promissory discourses of deeper embryo knowledge and expanded selection standardization, despite professionals having no conclusive evidence that TL improves pregnancy rates. Our research explores the use of TL tools in embryology labs. We pay special attention to standardization efforts and knowledge-creation facilitated through TL and its incorporated algorithms. Using ethnographic data from five UK clinical sites, we argue that knowledge generated through TL is contingent upon complex human–machine interactions that produce local uncertainties. Thus, algorithms do not simply add medical knowledge. Rather, they rearrange professional practice and expertise. Firstly, we show how TL changes lab routines and training needs. Secondly, we show that the human input TL requires renders the algorithm itself an uncertain and situated practice. This, in turn, raises professional questions about the algorithm’s authority in embryo selection. The article demonstrates the embedded nature of algorithmic knowledge production, thus pointing to the need for STS scholarship to further explore the locality of algorithms and AI.

## Introduction: Embryo Assessment, Algorithms, and Knowledge Standardization

Time-lapse (TL) imaging technologies were developed in the midst of a research-intensive period in the field of assisted reproductive technologies (ART). Scientific developments in the area of embryo diagnostics are a result of stagnating success rates in fertility clinics—rates that have only improved slightly since the 1980s (Gleicher, Kushnir, and Barad 2019). Although many seemingly “good-quality” embryos are transferred every year to patients, about only 30 percent of them result in a live birth, with minor variations by age, country, and clinic (Kupka et al. 2014). TL was designed to mitigate uncertainties surrounding scientific knowledge of embryo quality and implantation potential (Adjuk and Zernicka-Goetz 2013; Montag, Toth, and Strowitzki 2013; Schoolcraft, Meseguer, and The Global Fertility Alliance 2017). TL’s ability to facilitate closer monitoring of embryos is supposed to result, in theory, in a more efficient selection process. Although the current evidence on TL’s ability to improve pregnancy rates is not conclusive, clinic laboratories across the UK have incorporated imaging techniques in their work. Our research shows how TL imaging and algorithmic practices produce local outcomes as a result of complex human–technology interactions. Thus, the initial promissory discourses of algorithmic streamlined knowledge are transformed through lab practice. The technology has required professionals to rearrange lab routines, while also navigating the demands of the algorithmic embryo selection process. We highlight how algorithmic knowledge is not simply added through the introduction of a new technology. The knowledge is rather coproduced alongside the practices that the introduction of TL disrupts. Consequently, lab professionals navigate algorithmic choices they perceive as subjective, thus questioning the role of TL as an authoritative knowledge source.

Knowledge production and standardization in medicine have been topics of great interest for STS scholars (Cambrosio et al. 2006; Knaapen 2014; Moreira 2007; Timmermans and Epstein 2010). However, the ways in which standards are adopted or rejected in laboratory work (Doing 2004, 2008; Cetina 1995, 1999; Latour 1983) remain underexplored. Additionally, medical knowledge is increasingly reliant on algorithmic technologies, thus complicating existing relationship between knowledge-creation and laboratory practice. As new algorithmic technologies are introduced in biomedical contexts, it is also vital to probe the intersections among laboratory work, standardization, and the messy heterogeneous ways in which algorithms operate (Liu 2021; Ziewitz 2016). This article offers insight on these intersections by contributing both to emerging STS studies of the effects of algorithms and to the literature on medical knowledge production more widely.

In a review of studies on standardization, Timmermans and Epstein (2010, 69) call for a “careful empirical analysis of the specific and unintended consequences of different sorts of standards operating in distinct social domains.” Although the rise of knowledge standardization can be observed in many fields, healthcare in particular, has been undergoing a large standardization movement driven by the adoption of evidence-based-medicine (EBM) protocols in the past three decades. This has resulted in a focus on practice guidelines, increased standardization of outcome measures, and numerous meta-analyses medical literatures (Greenhalgh et al. 2008; Knaapen 2014; Moreira 2007; Timmermans and Berg 2003). The drive toward standardization, however, has also revealed the limitations of EBM. Clinical decision-making is still a complex process where professionals often make decisions based on local knowledge and experience (Berg 1999; Greenhalgh et al. 2008). As STS scholars grapple with the social implications of algorithms (Crawford 2016; Lee and Helgesson 2020), we offer a view of algorithmic lab technologies as situated and disruptive, suggesting that knowledge-creation through algorithms is a local process-in-the-making rather than a straightforward achievement through the introduction of a technology alone. More widely, we also suggest that scholars pay attention to the ways in which algorithmic technologies rearrange scientific practice.

## Embryo Knowledge and TL

Knowledge of human embryos remained limited until the late 1970s when ART began to be incorporated into medical practice (Chapko et al. 1995). Embryologists now know that optimal conditions can easily be disturbed when embryos are outside incubators. This creates tensions between the need to observe their development and the need to preserve them for successful implantation. Over time, embryologists developed a system where embryos are taken out of incubators for microscope observation only at specific points during their development, keeping disruption at a minimum. Before the introduction of TL, morphology (or how an embryo “looks”) was considered the best indicator of pregnancy potential (Holte et al. 2007). Although some studies indicate a correlation between “good-looking” embryos and pregnancy rates, there are exceptions to this rule (Meseguer, Kruhne, and Laursen 2012). Morphological assessment is a practice that continues due to tradition rather than robust evidence (Holte et al. 2007). It is sometimes categorized as “subjective” due to inconsistencies between embryologist observations (Bendus et al. 2006). Social scientists (Helosvuori 2019) note that embryo assessment is achieved through the combination of several factors, including lab practices and professional expertise.

The introduction of TL is intended to mitigate knowledge uncertainty about embryo potential (Kaser and Racowsky 2014). TL’s noninvasiveness coupled with the routinization of live-cell imaging (DiCaglio 2017; Landecker 2012) has contributed to growing professional interest in it. Moreover, TL can process large amounts of embryo development data. There is a growing literature in embryology that uses these data. Although few definitive conclusions have been drawn, the promise of further standardization in embryo selection has gained traction in embryology (Lundin and Park 2020).

In labs, TL use has introduced an additional criterion in embryo assessment: the timing of development events (e.g., nucleation, cell divisions). This facilitates a “morphokinetic” assessment of embryos that was associated with greater embryo viability in some exploratory studies (Meseguer, Kruhne, and Laursen 2012). TL also helps professionals detect abnormal events that can occur in-between standard daily microscope observations (Freour et al. 2012; Wong et al. 2013). Certain abnormal embryo “behaviors” (e.g., direct cleavage, where a cell divides into three very quickly) are associated with lower implantation potential (Liu et al. 2014; Rubio et al. 2012). The continuous embryo monitoring that TL provides is facilitated by incubator cameras that take pictures every five to twenty minutes, resulting in a detailed development video. TL software, however, is not yet able to detect developmental events automatically/through the use of AI. Rather, embryologists are required to annotate these and record information in the software so it can be processed by TL algorithms. Annotation involves embryologists watching each embryo video very closely and marking the exact timing of embryo developmental events. When annotating, embryologists register these developmental events with a time-stamp in the software. As such, they have to indicate exactly when the embryo reaches a particular stage, for example, the appearance of the nucleus, the nucleus fading, “cleavage events” (cell divisions), and various embryo expansion stages (e.g., morula, blastulation). The later stages of development are particularly hard to pin down precisely, according to embryologists. This is because cells may often appear fuzzy or overlapping.

Within social studies of IVF, embryos themselves have been a contentious object in scientific research and practice (Ehrich et al. 2007; Parry 2006; Scott et al. 2012; Svendsen and Koch 2008; Van de Wiel 2018, 2019). Embryos are a locus of uncertainty in medical knowledge (Parry 2006; Scott et al. 2012) in addition to being entangled with moral debates regarding the beginning of life and the ethics of disposal (Ehrich et al. 2007; Svendsen and Koch 2008). In this article, we focus on embryos’ implantation potential and how this is assessed scientifically with the help of TL tools.

## Working with TL

Firstly, TL technologies consist of an incubator with cameras (optical microscope) incorporated into its chambers (where the embryos are stored). One exception to this is the PrimoVision brand that consists of a camera that can be attached to petri dishes in a standard incubator. However, we found most labs prefer the cameras to be incorporated. Embryoscope is a particular brand that preferred by UK professionals. Secondly, TL technologies incorporate a software that allows the viewing of embryo images/videos on computers. The software also incorporates the embryo selection algorithm that draws on the staff data input. Although algorithms can differ and are customizable, their common purpose is to provide an embryo grade. This can be used in conjunction with morphological assessment to determine embryo implantation potential.

Selection algorithms may differ slightly across clinics. One of the early TL systems named Eeva was marketed as an AI-powered algorithm predicting which embryos are unlikely to become a viable blastocyst (Kaser and Racowsky 2014). However, studies increasingly show that universal selection algorithms are unlikely to work, as clinic populations vary. As such, in-house personalized algorithms are preferred (Fischer 2015). However, developing a custom algorithm requires large data sets that not all clinics have yet. The Embryoscope TL machines observed were often used in conjunction with a patented algorithm package named KIDScore (Known Implantation Data score) used to (1) deselect embryos that behave abnormally, (2) predict likelihood of implantation on day 3 and on day 5, and (3) enable clinics to develop their own algorithm following the collection of sufficient data on their patients outcomes. Clinics observed use KIDScore (although in different ways), while also building their own custom algorithm. If clinics use a different TL system (such as PrimoVision or Eeva), a different annotation system and algorithm are also used. However, these systems are significantly less popular.

## Data and Methods

The data included in our analysis are part of a project studying the impact of imaging technologies in IVF. These include relevant medical guidelines and policy documents, lab observations, and interviews with professional staff involved who have used TL. Professional observation and interview data were collected between June 2017 and March 2019. Detailed ethnographic observations were carried out by the authors in five England NHS sites (named here A, B, C, D, and E) where fertility treatment is provided. We observed lab routines and shadowed embryologists at each site for a minimum of three working days. We paid close attention to the use of TL in the lab, the annotation and selection process, and professional engagement with selection algorithms and information generated through TL technologies. The observations amounted to a total of 230 hours. Firstly, clinics were selected based on daily lab use of TL. Selection was also based on their availability and willingness to participate in the study. All five clinics agreed to participate and staff were informed in advance about study procedures, with all those observed signing a consent form prior to the start of our research. The study received university ethics approval as well as ethics clearance from the NHS and each clinic site. Following observations, professionals were approached by the authors regarding interviews. As with observations, interview participation was voluntary and involved the signing of an additional consent form. We conducted a total of twenty-five interviews. A small number of interviewees (e.g., clinic directors, nurses) were not lab staff but had relevant TL knowledge or had talked to patients about its use. The majority of those interviewed and observed are, however, embryologists. The interviews lasted between forty-five and ninety minutes were audio recorded and then professionally transcribed. Our questions focused on participants’ lab and work experiences, the challenges and benefits of using TL, and the technology’s place in IVF treatment.

TL has been consistently marketed on the basis that it can lead to improved rates of pregnancy. This has been a definite factor into labs adopting it, but staff are also aware the technology might not live up to its promise. Although clinic workers in our sample expressed that technological hype and competitiveness in the IVF sector contributed to their adoption of TL, they also stressed that they do not heavily market TL to patients and do not charge extra for it to be included in individuals’ treatment. For this reason, commercialization issues did not feature prominently in our data. Such issues have been explored in previous work (Van de Wiel 2018, 2019), and it is beyond the scope of this article to deal with them. In the analysis below, we focus on how the technology has been rolled out locally in UK labs and how knowledge creation is negotiated in practice.

We analyzed the data using grounded theory principles (Glaser and Strauss 2017). We started with a set of initial codes based on the TL literature, then developed refined codes and grouping categories as the research progressed. The authors constantly compared notes and observations that emerged from different research sites. The situated practices that emerged from the data reveal that the use of TL is contingent upon specific local procedures that problematize the TL standardization narrative. In the next section, we discuss the “locality” of TL practices and uncertainty as they relate to annotation, the algorithm and TL score use, as well as the sharing of TL embryo images with patients.

## Annotating Embryos: The Creation of New Lab Routines

The manual annotation process is a necessary precursor of TL algorithm output. One resulting critique of TL is the increased need for professional consensus on how to annotate. Annotating embryos is especially time-consuming. Thus, lab routines need to adjust for this additional work created by the introduction of TL. The length of time required to annotate varies depending on the quality of the embryo and professional experience. It also depends on how many embryos a patient has. For example, it is common for a patient to have five to ten embryos developing in the lab and sometimes more. For confident embryologists, the process can be quicker. However, our observations revealed that consulting with other lab staff to reach consensus on difficult annotations is part of the process and needed at least occasionally. We observed different annotation routines in each clinic, with each having to rearrange their practices to accommodate TL use.

Firstly, clinics can make different choices regarding which embryos to annotate fully, from fertilization to day 5. Embryologists at the biggest clinic (D) in our sample decided that annotating all embryos would create unmanageable workloads, as annotating all could take several hours daily for at least two staff. The embryos that are not annotated are usually those that die early and are discarded. In some cases, only the ones that are good candidates for transfer are annotated fully. In such cases, the goal is to have as much information about these as possible, rather than collect data on all embryos. The lab director of a smaller clinic (B) and TL-use advocate, however, stressed that, for her, it is important to annotate all embryos in order to take advantage of all data they provide. Consequently, she encourages staff to annotate all, time permitting. This is possible when clinics have a manageable volume of patients. In clinic B, we observed staff using time outside egg collection and transfer windows to catch up on annotations. In all labs, we observed a preference for annotating either early in the morning before egg collection procedures or later in the afternoon after patient appointments.

In most clinics, annotation training is still ongoing. Some sites had a couple of staff specifically tasked with embryo annotations. Others, however, annotate more widely and conduct regular in-lab quality control exercises. To maximize TL benefits, embryologist agreement on annotation points is needed. Nonetheless, during interviews, embryologists repeatedly emphasized that some stages of embryo development might be harder to identify, thus leading to “subjective” opinions on annotations. Depending on the level of TL integration, we observed more streamlined annotation consensus procedures in three of our clinics (B, C, and D). However, integration often came after an arduous training process. Importantly, embryologists talked about the changing scientific consensus:

Well, it completely changed how you work as an embryologist. It was so…. And I, we got it when I was mid-training so I’d gone from one way of doing it to oh no, now you need to learn it a completely new way. *And the annotation is constantly changing. There’s new things that we have to learn how to annotate, definitions are changing, the consensus is changing all the time.* And so I do remember being very, very late in the lab quite a lot trying to get my head round how to annotate and what to annotate and yeah, it being quite difficult. And there’s still people that struggle with it now. You know, that definitely people find it really difficult. And also to see what the point of it is. You know, we annotate about over forty things on one embryo and we use a handful of them. So it has definitely increased workload. And yes, my experience it was at the beginning very frustrating because it was a lot of, a lot more work for what, for what benefit. And then I think that’s what started me off on the well, there has to be a reason why we’re doing this. (Lab director, Clinic B)

The lab director establishes the connection between TL-led changes and the uncertainty surrounding annotation standardization. Various staff talked about the tediousness of keeping up to date with medical literature developments. To a certain extent, TL has introduced another learning curve in the lab, especially for those who were training when the machines became popular. There is an optimistic caution in the professional community that TL integration will deepen embryo knowledge. However, connecting this knowledge to lab practices requires additional professional engagement with the scientific literature.

We found that staff on the ground have to confront many questions regarding consistency and quality control in TL practices. An embryologist at clinic A, a clinic that has not yet fully integrated TL explained:

So at the moment there’s only one or two people annotating all of the embryos that are put into the Embryoscope because they have been trained and they, their annotations have been compared to make sure that they’re similar or the same. So at the moment we are trying to train everyone to do be able to do annotations but it’s difficult to have, you know, a very cohesive, a very…[interviewer: consistent?] Yeah, consistent, that’s the word I’m looking for. Consistent annotation. For things like cell divisions it’s fairly simple because you can see when it’s divided or not but things like time to blastulation or time to the start of, start of blastulation, so as soon as you can see a cavity appearing that’s a little bit subjective. Even with one operator it can vary but, or between embryos you can, your annotation time might vary slightly so in that sense it does increase the workload slightly having the Embryoscope in there. (Embryologist, Clinic A)

With TL, labs have to create infrastructures for annotation quality control. As highlighted above, some aspects are perceived as more subjective and thus in need of standardization (Timmermans and Epstein 2010). Objectivity, defined by staff as consistency in annotation, was seen as an important pursuit meant to facilitate optimal use of TL. We observed staff completing quality control exercises on a couple of occasions. Junior embryologists questioned their annotations more, indicating that, to a certain degree, such skills are picked up through repeated practice only. When asked what happens with inconsistent annotations, most staff said that these are discussed with the person in charge of the exercise, in a process where they assess why one person’s annotations deviated significantly from expectations set by senior staff. Labs also used a UK-specific external quality control exercise operated through the National External Quality Assurance Service (NEQAS). TL videos are a relatively new feature for this service. Most staff said that the quality of the TL videos provided by NEQAS was not as good as the labs’ own. The NEQAS exercise was usually managed in a similar way to the internal exercise, where one senior member of staff was in charge of discussing inconsistencies. Although such quality control exercises exist, staff stressed uncertainty in light of the need to introduce new lab practices and rearrange how quality control for annotation is accomplished.

## Algorithms-in-the-making: TL and the Complex Human–Technology Interplay

Our data reveal that TL is not a straightforward technological solution to standardizing and mainstreaming embryo knowledge. In this section, we suggest that TL and algorithmic lab technologies more broadly require careful unpacking, given their need to be activated through professional input. Lab engagement with TL algorithmic platforms differs and is very much dependent on lab expertise and willingness work on adapting software to lab practice. The use of TL has exposed the need for additional expertise in the area of biostatistics—expertise that is not typically built into fertility care. Nonetheless, labs across the UK that have adopted TL tools and have embraced the learning process required, although with different degrees of enthusiasm. For example, in our sample, we found that at least two labs had overall reservations regarding the benefits of using TL. Uncertainty regarding optimal use of algorithms featured prominently in our discussions with embryologists. Staff were highly aware that, although the technology holds promise, significant input was needed from them in order for the algorithm to function at its full potential. This included annotation as well as setting up algorithm parameters and embryo score outputs.

In the initial stages of adopting an Embryoscope TL machine (used by all labs observed), staff need to set up the KIDScore algorithm offered as an option for an extra cost. The usage of the KIDScore package was seen by most as a practice in need of adapting to their own clinic’s needs. Algorithms developed outside of the clinic were often viewed with suspicion:

What I think might happen is that the undisturbed culture will maybe help. But then again I don’t know, I just don’t know if we’re using KIDScore to its full capacity for it to actually make a difference. And I don’t think anybody knows enough about KIDScore and enough about the algorithms of embryos to actually say yeah, this is what you need to select the best embryo. I don’t really trust it that much, that algorithm. I do it because it’s like we have to do it and whatever but it’s very rare that we actually get a higher KIDScore on what we would have thought was a lower quality embryo so usually they kind of match up so I don’t know, I don’t know how much. And it’s also very subjective, KIDScore. You know, you’re talking about when I think it’s expanded and like you or someone else thinks it’s expanded and it can be completely different. So I think it’s subjective too. So I don’t think it’s like…I think again the undisturbed culture and the idea of being able to look at it and you know, and you can see reverse cleavage and stuff, that’s quite interesting. But I don’t know if it makes a big difference. (Senior embryologist, Clinic B)

The algorithmic black box creates knowledge uncertainty for lab staff that are not directly involved with its creation or adaptation to their own clinical practice. This uncertainty was perceived by participants as “subjective” knowledge, which they contrasted to an objectivity ideal (or standard) that TL was meant to achieve. The need for extended human input into TL was perceived as a source of subjectivity, thus problematizing the promise of TL as a technology that could ensure a more seamless embryo selection process. Labs with research-active embryologists who could coordinate the use of algorithm data were more confident with using TL. However, such practices create new skillset needs for some embryology labs. Despite the promise of TL, its successful implementation on the ground depends on new expertise. As the respondent above stresses, the annotation input in algorithms can still be categorized as a “subjective” endeavor as it requires embryologist consensus on visual data (e.g., the start of embryo expansion) and is not automated. Thus, the management of uncertainty in relation to TL use includes many variables, from the systematization of annotation procedures to the setting of algorithm parameters. The constant need for human input into the machine was seen as a main source of this uncertainty, thus rendering the technology somewhat incomplete in the eyes of our participants.

Uncertainty in local practice and technology–staff interactions was managed to some extent by embryologists with TL research expertise. Two labs had at least one member of staff with significant knowledge of TL algorithms. Nonetheless, the algorithm options seemed daunting for most. For example, if a lab decides to build its own algorithm, there are a multitude of directions to take with the annotations included, the weight given to different variables and the inclusion of different patient conditions. This coupled with the constant need to refine the algorithm through the collection of new data. In small clinics, staff were weary of the long transition to a robust algorithm, all the while knowing that it might not lead to increased pregnancy rates. The uninterrupted incubation aspect of TL and the images generated through it seemed to be more tangible benefits when compared to the uncertainty of algorithms and their outputs.

An illustration of the choices and human work involved into building TL algorithms can be seen below. During lab observations, an embryologist from clinic C explained their annotation and algorithm-building process:

The senior embryologist says that Embryoscope has a variety of options for grading embryos, but they only use the one overall grade at the top. Otherwise, it would become too complicated—they don’t think there is a need to bother with all options for grading. She emphasizes that their choices on what to annotate are based on their own data. She also stresses that they used the medical literature to help them decide what is important to look for in terms of annotating and embryo development. The embryologist stresses that this particular model that they use could not be used in a different lab because it is based on their data and also based on the media that they use. They’ve been using the same one for approximately two years now. She says they are happy with the current model, but they could change it if they wanted to. However, this cannot be done anytime, on the spot. It requires special permissions to set up and should be done outside of the working day. Also, they are the ones who decide how much weight to give certain embryo events. In the table of event scores they look at, I find out, the weight assigned was determined by the lab staff. Therefore, even though TL gives them an embryo score, it is determined by how they programmed the events to be weighed. (Author observation notes, Clinic C)

Clinic C put significant effort into building a systematic TL process. However, not all clinics are able to invest the same amount of time and effort into algorithm-building. Research and knowledge of statistical parameters are necessary to make optimal choices. All clinics emphasized how their use of TL is particular to their situation and their expertise. Thus, how TL is integrated largely depends on a seamless integration with the professional capabilities of the lab where it is used. This discourse countered the wider enthusiasm for knowledge standardization in embryo assessment through the use of TL. The study of local lab practices (Latour 1983) reveals all the different ways in which TL can be used on the ground. We noticed different levels of engagement with the algorithm functions of TL. Ultimately, staff always have the option of using it simply as an incubator that generates embryo images. However, this was not seen as cost-effective given the high cost of TL technologies. Ultimately, under the current scientific lack of consensus around morphokinetic assessment, it is clinics that choose if they want to engage in the process of algorithm creation/adaptation at all.

## Choosing Embryos: Algorithmic Output and Questions of Expertise

Although TL algorithm scores are meant to improve embryo selection, we found that this new knowledge dimension was not always easy to integrate within established professional practices. Embryologists worked to incorporate this new technological expertise into their routine, but also questioned the algorithmic output and how it might pose a challenge to professional expertise. During observations, we studied how TL algorithm scores are produced and featured in clinical embryo transfer decisions. The types of scores produced by lab algorithms varied slightly. For example, clinic D had a score that could go up to 75, while clinic B had a score between 1 and 6. Others were receiving a letter grade output from the TL algorithm. It is usually the highest score that indicates a good-quality embryo. Transferring more than one embryo is discouraged in UK clinical practice with some exceptions, making the task of choosing only one difficult, especially when there are several of good quality.

All clinics showed resistance to relying on TL scores exclusively when choosing transfer embryos. Morphological grading is entrenched in decision-making. We observed decisions being made on a case-by-case basis, according to professional judgment (Greenhalgh et al. 2008). TL scores were sometimes viewed with skepticism or even ignored:

The embryologist thinks that the score 65 embryo looks better than one with a score of 70, which are meant to be “better” according to the TL algorithm. She looks at the annotations and some annotation scores appear in red when she looks at them in the table, meaning that the event did not happen within the time expected. She wonders if maybe one of the staff did not annotate this properly. She suspects that maybe a minor mistake was made because, to her, the 65 embryo looks better and she would choose it over the higher scoring one. It is interesting that she attributes this to staff error rather than program error. Also, the score doesn’t seem to make her question her own judgment. (Author observation notes, Clinic C)

Here, the embryologist makes a judgment on embryo quality based on morphology. For her, this overrides the TL score, which she suspects is lower because of erroneous annotation. We witnessed a few such instances where morphology or the “old scoring system” was prioritized when choosing an implantation embryo. Often, staff felt more confident in the established way of choosing embryos. This is not to say that embryologists do not care, more generally, about TL data. It rather shows that standardizing the incorporation of new information is rather difficult. Additionally, without clear evidence on the benefits of TL for pregnancy rates, embryologists viewed the new scores with skepticism. Staff were aware of the subjective dimensions of annotation and algorithm-creation. In the example above, a senior embryologist questioned the annotation process rather than her own judgment on the morphology of the embryo.

Depending on their confidence regarding the TL score’s robustness, professionals expressed being interested in using these scores. Their desire to do so, however, was limited to situations where many embryos of similar quality are available to choose from. Interestingly, rewatching the TL videos helped embryologists reevaluate an embryo, if necessary. This enhanced confidence in transfer choices, independently of the algorithm feature. In our notes from clinic B, we wrote:

I ask the embryologist what helps her decide if she’s unsure which embryo to pick. She says she watches the videos side by side very slowly and looks for small anomalies (fragmentation %, for example she says) and only after that she will look at the score that TL gives them. But she adds that the score should always be taken “with a grain of salt.” She explains that they don’t use it all that much (it is rarely necessary she seems to suggest). She’s glad this patient’s got many good embryos, but she says she won’t need that many. (Author observation notes, Clinic B)

Here, the embryologist reinforces the need to be skeptical about TL scores. As we suggested above, this raises questions about the possibility of TL score standardization and the algorithm’s authority in choosing embryos. However, as we already explained, algorithms can vary widely between clinics. Consequently, embryologists see TL integration as a local work-in-progress rather than knowledge passed through top-down standardized guidelines (Knaapen 2014).

We were also interested in how TL scores were deployed outside of the lab. TL scores were not usually discussed with patients, as they were seen as an element that might cause confusion. Furthermore, with no clear consensus on how to interpret them, staff felt it would be unwise to overemphasize these to patients. Scores were recorded by some clinics on patient forms, but more often than not, patients were only given the morphological embryo grade. As this classification system is more established, patients are able to search information about it online and decipher what it might mean in relation to implantation rates for that grade. As TL scoring can vary between labs, patients would find it harder to find relevant information on these scores. Nonetheless, embryologists explained to us that they do refer to TL videos in their conversations with patients as this allows them to explain what they have seen the embryo “do.”

TL facilitates the travel of embryo information outside of the lab. Thus, it creates the possibility, according to staff, of patients questioning their expertise and decisions on which embryos to transfer. Three clinics (B, C, and D) offered patients the option to have a USB stick with the TL video of their implanted embryo. This option was not taken up very often, but some staff felt it could be better advertised. Regardless, the videos were usually shared only after a pregnancy was confirmed. Two clinics (A, E) in our sample avoided sharing TL images and videos, unless the patients brought this up themselves. Although not all, some embryologists felt anxious about the possibility of having their expertise questioned if patients share TL videos with others who might provide a second opinion on their embryos. Consequently, sharing TL information, including potential access to an embryo livestream was often seen as an opportunity for the undermining of scientific expertise. This view resonated with many professionals:

I think that’s a difficult one because again it’s their information but the problem will be is it’s a very subjective field looking at embryos and you know this better than I do, I’m not a scientist, but it still subjective, there will still be some people that will still grade embryos slightly different to others although you have a pathway and follow protocol, there will be a slight variation and my worry is will people use it then and it has a negative effect. I want to take this to somebody else for a second opinion. And I think that’s the only danger I see. Not that I don’t think somebody should have a second opinion but it’s a very, a subjective assessment and I know embryologists have pretty much now a standardization for grading of embryos but I still think that could happen. And taking that now to a private independent embryologists and you know, I just worry about the integrity of that. But I think it’s powerful information, powerful but it’s theirs, you know, it is their information but I just think it’s powerful information that could be used sadly not always used in the right way. (Senior fertility nurse, Clinic B)

Interestingly, our respondent emphasizes the limits of current standardization as it exists. She talks about the standardized morphological grading as it has been used for the past decades and contends that, even there, she sees issues around scientific objectivity. To a certain extent, the existence of TL and its functions threaten to disrupt the current order: the technology exposes patients to what was largely “inside knowledge” before. As such, it becomes evident that underneath the surface of TL’s algorithmic promises, lie uncertainties regarding the best use of this technology and information generated through it. Enthusiasm for sharing TL videos with patients varied significantly during our conversations with staff. Not only did each clinic have different ways of providing patients with information; each member of staff also had differing views on whether or not the process is beneficial for patients at all. The embryo information that can be retrieved through TL was generally seen as having the potential to make patients even more anxious about a process that is already challenging.

## Conclusion

We have outlined above how local algorithmic practices coexist in tension with standardization expectations. Through the introduction of TL in IVF labs, professionals have had to adapt to the demands of this new technology. The perceived subjective input that TL requires deems the technology as an incomplete entity—an entity whose authority professionals challenged periodically, while also working to improve algorithmic output. Through the exploration of the TL case, we argue that biomedical algorithmic knowledge coexists in tension with complex lab routines and clinical contexts. This is partly a result of the input needed from professionals to make the technology “work” and the questions staff raise about perceived subjective practices. STS scholars often conceptualize technologies as situated (Aviles 2018; Coutard and Guy 2007). In the context of increased interest in the social life of algorithms (Ziewitz 2016), we suggest that algorithms themselves can also be conceptualized as situated practice. Moreover, we add evidence that actors may struggle with valuing algorithmic technologies (Lee and Helgesson 2020) and that algorithm effects cannot be anticipated in a predictable manner (Neyland 2016). As seen in the TL case, the local embeddedness of algorithmic practices impacts knowledge creation in ways that standardization efforts do not necessarily anticipate prior to the introduction of the technology.

Our findings show that embryologists working with TL are faced with numerous decisions in relation to annotation processes, algorithm implementation, TL score use, and how to share TL information with patients. The analysis illuminates lab practices, thus complementing previous studies of embryo selection (Helosvuori 2019), professionals’ negotiation of EBM standards in relation to TL use (Perrotta and Geampana 2020, 2021), and of TL commercialization (Van de Wiel 2018, 2019). In local practice, uncertainties around professional algorithmic input lead to TL disrupting and rearranging professional practices, rather than straightforwardly resolving uncertainties in embryo knowledge. We contend that consensus and standardization in embryo assessment are ever-evolving processes, and that TL has added increased complexities to this process rather than having simplified it. Thus, we suggest that STS scholars pay attention to the disruptive qualities of algorithmic technologies as they are used in biomedicine. We also suggest that the degree of human–machine interaction required by such technologies greatly shapes how they are perceived by professionals. TL has raised questions about the authority of algorithmic outputs and highlights how professional judgments feature the subjective/objective dichotomy, where objectivity is associated with knowledge standardization and certainty, while subjectivity is associated with a high level of human involvement.

More broadly, our case study makes an important link between professional movements encouraging increased knowledge standardization through algorithmic technologies and the actual implementation of such standards (Greenhalgh et al. 2008; Knaapen 2014; Moreira 2007; Timmermans and Berg 2003). As others have shown (Greenhalgh et al. 2008), clinical decision-making is still a process that entails complexities that professionals have to navigate based on local knowledge and their previous experience. Algorithmic standardization, in particular, we suggest, is a process-in-the-making where the introduction of AI-based technologies does not automatically lead to a straightforward generation of knowledge. As such, we stress the need to study algorithmic lab technologies at the local level to understand (1) how they reshape medical practice, (2) how the interplay between professional practice and such technologies shapes biomedical knowledge, and (3) how algorithms and their output are incorporated and/or resisted in clinical practice. Our findings draw attention to the embedded nature of algorithms and the local work that sustains them. We suggest that future STS research agendas on AI and algorithms need to further probe the contingent nature of such technologies by asking how “algorithm work” is done in practice and paying closer attention to interactions between human actors and algorithms.
